# The Surprising Story of Fusicoccin: A Wilt-Inducing Phytotoxin, a Tool in Plant Physiology and a 14-3-3-Targeted Drug †

**DOI:** 10.3390/biom11091393

**Published:** 2021-09-21

**Authors:** Mauro Marra, Lorenzo Camoni, Sabina Visconti, Anna Fiorillo, Antonio Evidente

**Affiliations:** 1 Department of Biology, University of Rome Tor Vergata, Via della Ricerca Scientifica, 00133 Rome, Italy; mauro.marra@uniroma2.it (M.M.); camoni@uniroma2.it (L.C.); visconti@uniroma2.it (S.V.); anna.fiorillo@uniroma2.it (A.F.); 2 Department of Chemical Sciences, University of Naples Federico II, Complesso Universitario Monte S. Angelo, Via Cintia 4, 80126 Napoli, Italy

**Keywords:** fusicoccin, diterpene, phytotoxin, bioactive metabolites, biosynthesis, structure-activity relationships, plasma membrane H^+^-ATPase, 14-3-3 proteins, protein-protein interaction, drug design

## Abstract

Fusicoccin is the α glucoside of a carbotricyclic diterpene, produced by the fungus *Phomopsis amygdali* (previously classified as *Fusicoccum amygdali*), the causal agent of almond and peach canker disease. A great interest in this molecule started when it was discovered that it brought about an irreversible stomata opening of higher plants, thereby inducing the wilting of their leaves. Since then, several studies were carried out to elucidate its biological activity, biosynthesis, structure, structure-activity relationships and mode of action. After sixty years of research and more than 1800 published articles, FC is still the most studied phytotoxin and one of the few whose mechanism of action has been elucidated in detail. The ability of FC to stimulate several fundamental plant processes depends on its ability to activate the plasma membrane H^+^-ATPase, induced by eliciting the association of 14-3-3 proteins, a class of regulatory molecules widespread in eukaryotes. This discovery renewed interest in FC and prompted more recent studies aimed to ascertain the ability of the toxin to influence the interaction between 14-3-3 proteins and their numerous client proteins in animals, involved in the regulation of basic cellular processes and in the etiology of different diseases, including cancer. This review covers the different aspects of FC research partially treated in different previous reviews, starting from its discovery in 1964, with the aim to outline the extraordinary pathway which led this very uncommon diterpenoid to evolve from a phytotoxin into a tool in plant physiology and eventually into a 14-3-3-targeted drug.

## 1. FC: A Wilt-Inducing Phytotoxin

### 1.1. Discovery

**Figure 1:**
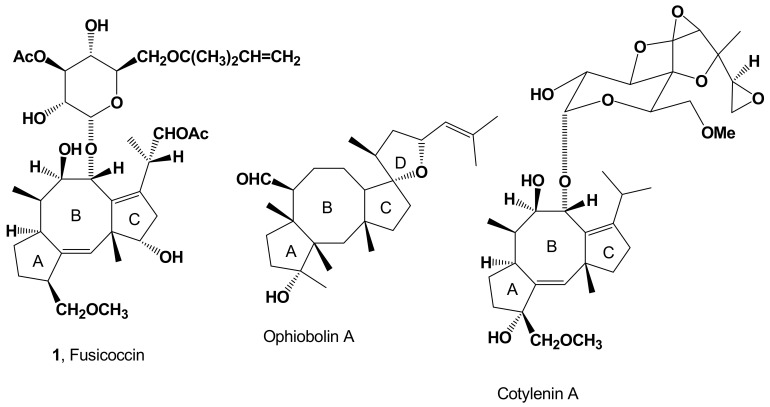
Structure of fusiccocin (**1**), ophiobolin A and cotylenin A, the main representative members of the three fusicoccanes-related families, sharing the same 5:8:5 carbotricyclic ring system.

Fusicoccin (FC, Figure 1, Table 1) is the α-glucoside of a carbotricyclic diterpene whose IUPAC name is (*S*)-2-((1*S*,4*R*,5*R*,6*R*,6a*S*,9*S*,10a*R*,*E*)-4-(((2*S*,3*R*,4*S*,5*R*,6*R*)-4-acetoxy-3,5-dihydroxy-6- (((2-methylbut-3-en-2-yl)oxy)methyl)tetrahydro-2*H*-pyran-2-yl)oxy)-1,5-dihydroxy-9-(methoxymethyl)-6,10a-dimethyl-1,2,4,5,6,6a,7,8,9,10a-decahydrodicyclopenta[a,d][8]annulen-3-yl)propyl acetate. Its current name was derived from that of the fungus producing FC, *Phomopsis amygdali,* previously classified as *Fusicoccum amygdali*. The history of FC started almost 60 years ago, when *P. amygdali* was isolated in the south of Italy as the causal agent of a devastating disease of almond and peach trees [1]. The disease’s symptoms are the formation of cankers on branches, as well as the chlorosis and necrosis of distal leaves, not yet colonized by the pathogen [2]. The effect on distal leaves led to the hypothesis that systemic symptoms could be due to the translocation to non-infected tissues of one or more phytotoxins produced by the fungus during infection. This hypothesis prompted studies aimed to their isolation from culture filtrates of *P. amygdali* and their successive chemical and biological characterization [3]. The main diterpene-glucoside isolated, together with several related minor metabolites, was originally named FC A. This compound was able to reproduce part of the disease symptoms, such as the wilting of leaves [2,4,5], due to its ability to induce irreversible stomata openings and consequent uncontrolled transpiration [5]. Successively, FC was isolated from green peaches artificially inoculated with *P. amygdali* and from almond shoots naturally infected by the fungus, definitely proving that FC is a vivotoxin [6].

### 1.2. Structure Determination

The structure of FC was elucidated for the first time in 1968 by the group of Alessandro Ballio at the Istituto Superiore di Sanità, (IT) [7], by degradative chemical and spectroscopic studies, while its absolute configuration was assigned by X-ray analysis of the 12-*O*-*p*-iodotosyl derivative [7]. Concordant results, using similar methods, were reported by Barrow et al. [8,34]. The very original carbon skeleton of FC is characterized by a 5-8-5 ring system, which is shared with other families of bioactive terpenes, such as sesterpenoid ophiobolins and diterpenoid cotylenins. Ophiobolins, originally isolated from *Ophiobolus miyabeanus,* a fungus pathogen of rice, maize, and sorghum, were the subject of a number of chemical and biological studies reviewed by Au et al. [41]. Cotylenines, isolated from the fungus *Cladosporium* sp. 501–7W, are diterpene-glucosides closely related to FC which display in vivo activities both in plants and animals similar to those of FC [36,37,42,43,44,45,46,47,48]. The structures of the most representative members of the two terpenoids families, ophiobolin A and cotylenin A, respectively, are reported in Figure 1.

Further chemical degradative studies concerning the nature of substituent groups of FC allowed the ability to ascertain the occurrence of one glucosyl, one methoxy, one acetoxy, and two hydroxy aglycone substituents. The sugar residue carries an acetoxy group at C-3 and a 1,1-dimethylallyl ether at C-6 [29,49]. The efficient incorporation of DL-[2-^14^C] mevalonic acid lactone confirmed that FC has a polyisoprenoid structure [8,29]. Successive chemical investigations also carried out by heavy atom incorporation in FC derivatives confirmed the structure assigned to the toxin [34].

### 1.3. Minor Diterpenoid Metabolites of Phomopsis amygdali

**Figure 2:**
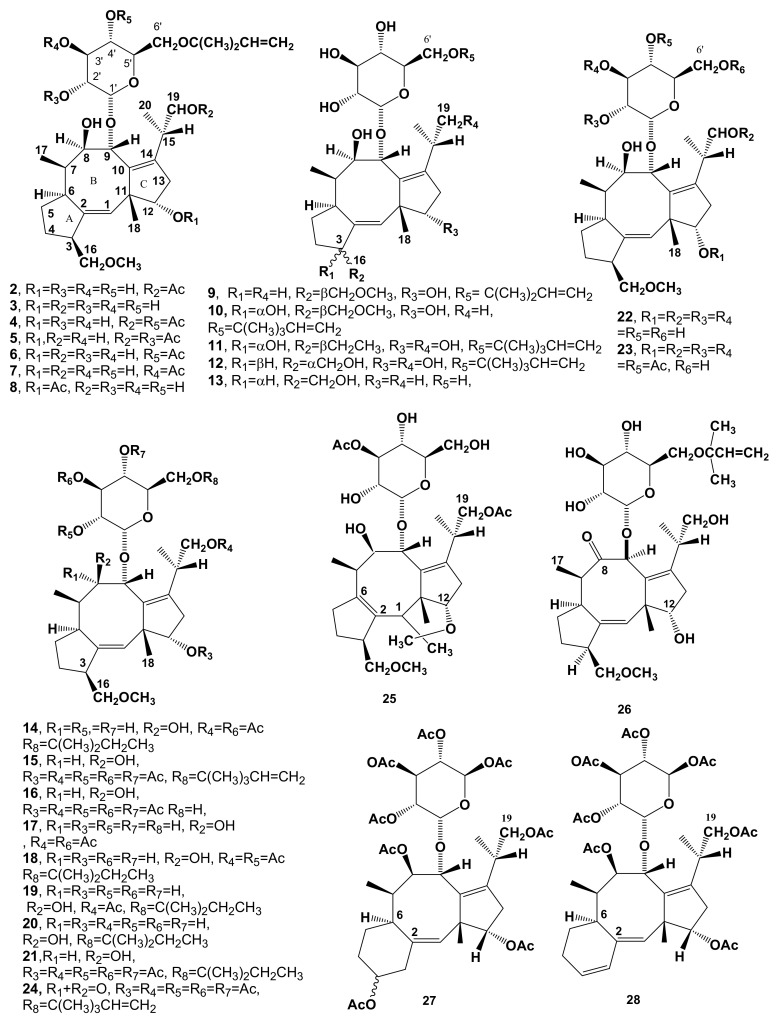
Chemical structures of FC major semisynthetic derivatives, and minor diterpenoid metabolites isolated from culture filtrates of *Pamygdali,* used in the biosynthetic and structure-activity relationship studies.

**Figure 3:**
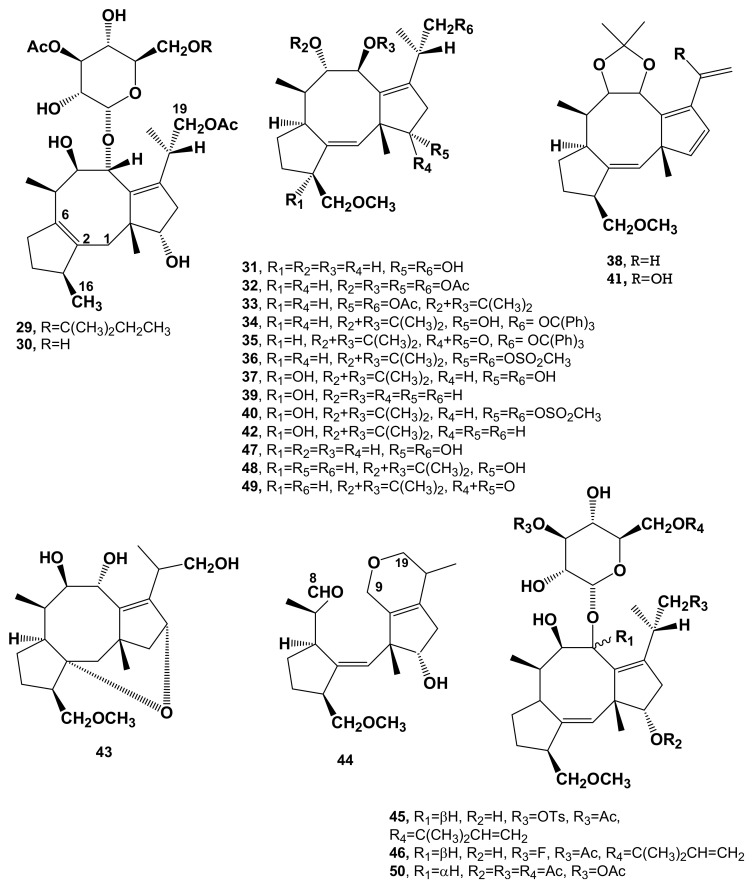
Chemical structures of other minor diterpenoid metabolites isolated from culture filtrates of *P. amygdali*, FC semisynthetic derivatives, and cotylenol, used in the biosynthetic and structure-activity relationship studies.

The remarkable biological activity of FC and its potential practical applications in agriculture prompted large scale productions by pilot plant level fermentation [50]. This production not only allowed the obtainment of hundreds of grams of FC but also consistent amounts of different metabolites, synthesized in minor abundance by the fungus and differing from **1** in the functional groups linked to both glucose and/or aglycone ANSWER: Okmoieties. Two metabolites, named monodeacetylFC, and dideacetylFC (**2** and **3**, Figure 2, Table 1), were firstly isolated from the mother liquors of FC crystallization. Compounds **2** and **3** were also prepared by the reaction of FC with diluted Na_2_CO_3_ and NaOH, respectively [9]. The iso- and allo-FC (**4** and **5**, Figure 3) are both isomers of FC, probably formed through the migration of the *O*-acetyl group carried at C-3’ of the glucose moiety to C2′ and C-4′, respectively [20]. They were also prepared by a reaction of FC with a pH 8.8 borate buffer at room temperature for 4 h [9,20]. Further chromatographic purification of the organic extract of the FC crystallization mother liquors allowed the isolation of the other three minor metabolites which are structural isomers of monodeacetylFC, namely monodeacetylalloFC, monodeacetylisoFC, and 12-acetyldideacetylFC (**6**–**8**, Figure 2, Table 1) [21]. A further minor metabolite isolated from the same organic extract was identified as 19-deoxydideacetylFC (**9**, Figure 2, Table 1). Its structure was confirmed by the synthesis through the monotosylation of dideacetylFC (**3**), followed by a reduction with LiAlH_4_ [22]. Metabolite **9** was also isolated from Barrow et al. [23] and named FC-J (19-deoxydideacetylFC), which was also synthesized from FC. C8-tritiated **9** was used in a feeding experiment carried out to demonstrate that it was a biosynthetic precursor of FC [23].

Other metabolites isolated from the same organic extract were: (i) the 3-α-hydroxy derivative of 19-deoxydideacetylFC (**10**, Figure 2, Table 1); efforts to correlate metabolite **10** to **9** failed, as the amount of isolated **9** was too low to allow crystallization and chemical derivatization [24]. Metabolite **9** carries a α-hydroxy group at C-3, shared by all cotylenins [36,37,45]; (ii) the 3-α-hydroxydideacetylFC (**11**, Figure 2, Table 1), whose structure was confirmed by the identity of spectral properties of its dihydroderivative, which showed a *t*-pentyl group instead of a *t*-pentenyl bonded at C-6′ as in FC, with those of the dihydroderivative of the product obtained by KMnO_4_ oxidation of dideacetylFC [25,26]. This reaction was previously optimized starting from the 8,9-isopropylidenedeacetylaglyconeFC (or acetonidedeacetylaglyconeFC, **33**, Figure 3, Table 1) to obtain the corresponding 3-α-hydroxy derivative (**37**, Figure 3, Table 1) and was carried out in order to investigate the C-3 as the site of incorporation of tritium radioactivity during the biosynthetic study, as below reported [26]; and (iii) 16-*O*-demethyl-19-deoxydideacetyl-3-*epi*-FC (**12**, Figure 2, Table 1) [27], whose structure was confirmed by X-ray crystallography of the corresponding aglycone [28].

A further minor metabolite was isolated by Barrow et al. [23] from the culture filtrates of *P. amygdali*, and identified by degradative studies and comparisons with FC and other minor metabolites, as 16-*O*-demethyl-19-deoxy-de-*t*-pentenyldideacetylFC, also named fusicoccin H (FC-H) (**13**, Figure 2, Table 1). A feeding experiment using C-8-tritated FC-H demonstrated that it is a biosynthetic precursor of **1**.

### 1.4. Biosynthesis

The study of the biosynthesis of FC was carried out in 1978–1982 by Ballio and coworkers [35,39,51,52] to answer the following questions: (i) is FC a “true” diterpene or a rearranged sesterpenoid? (ii) what is the mechanism of cyclization of the acyclic precursor? and (iii) what is the succession of reactions for the funzionalization of the carbotricyclic diterpene to yield FC?

The low level of incorporation (0.2%) did not allow the opportunity to study the biosynthesis of FC using NMR experiments after the incorporation of labelled mevalonic acid lactone (MVA). Thus, experiments of incorporation of MVA differently labelled with ^14^C and ^3^H, followed by chemical degradation, were performed [35,39,51,52] during in vivo biosynthesis. [2-^14^C]-DL-MVA-incorporating FC was degraded by treatment with a Fritz and Schenk reagent [52] into derivative **27** (Figure 2, Table 1), which lacks the *t*-pentyl residue. The specific radioactivity of the *t*-pentenyl residue incorporated into **27** was different by 1/4 of the total one of the corresponding aglycone, demonstrating that this residue bonded to the C-6′ of the glucose was independently biosynthesized and that FC is a diterpenoid and not a rearranged sesterpenoid. In the same reaction, the corresponding diene (derivative **28**, Figure 2, Table 1) was obtained as well [31].

**Figure 4:**
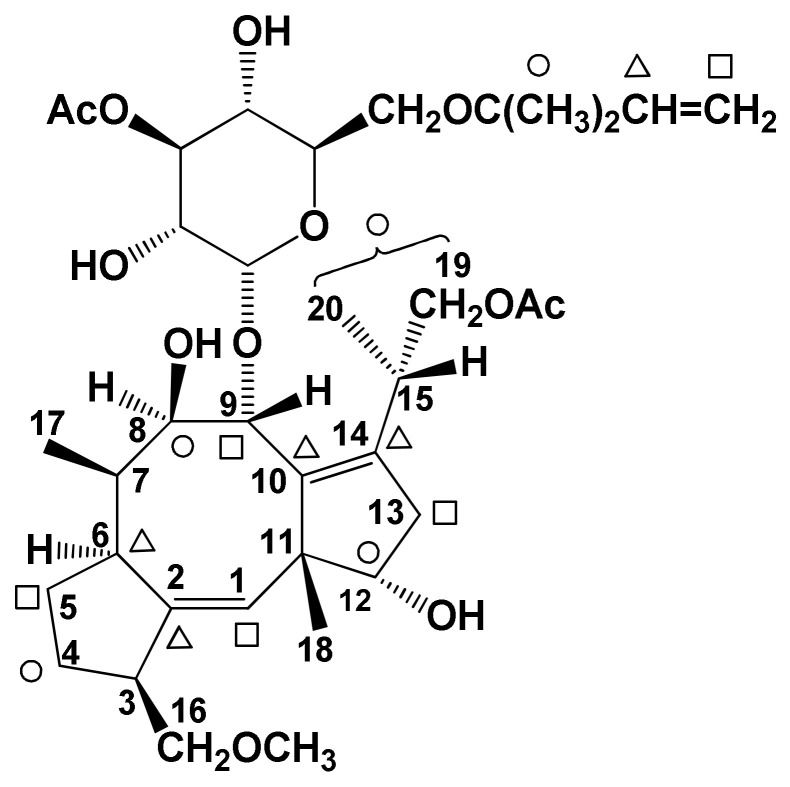
^3^H and ^14^C incorporation sites in radiolabeled FC. ○, ∆ and □ are sites of incorporation of [2-^3^H_2_, 2-^14^C]-, [(4*R*)-4-^3^H, 2-^14^C] and [5-^3^H_2_, 2-^14^C]MVA.

**Figure 5:**
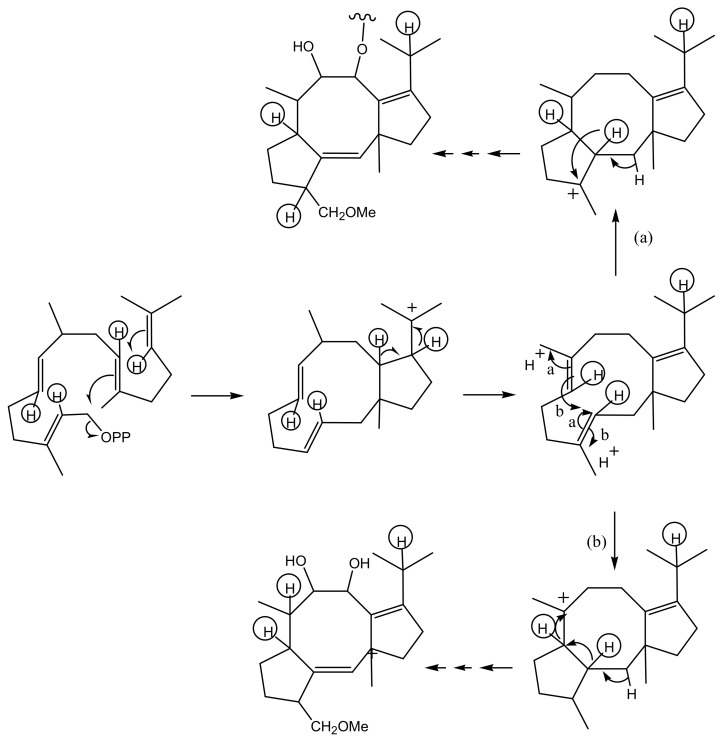
The two biosynthetic pathways of cyclization of GGPP into the characteristic 5:8:5 carbotricyclic skeleton of FC. Circled hydrogens arise from [(4*R*)-4-^3^H_1_, 2-^14^C]MVA.

The study of the mechanism of cyclization of geranylgeranyl pyrophosphate (GGPP) or its biological equivalent was carried out by the incorporation of three differently double-labelled MVAs, namely ([2-^3^H_2_, 2-^14^C]-, [(4*R*)-4-^3^H, 2-^14^C]-, and [5-^3^H_2_, 2-^14^C]-MVA, respectively. The theoretical positions of ^3^H and ^14^C atoms incorporated in **1** are reported in Figure 4. The radioactive FC obtained by the incorporation of differently labeled MVA was diluted with the non-labelled one, in order to have a sufficient amount of **1** to perform a chemical investigation of the incorporation site (Figure 4). The results obtained allowed ruling out the occurrence of a 1,5 shift of the tritium atom of 2-labelled MVA from C-8 to C-15, as previously observed in the ophiobolin biosynthetic pathway [32,53] and therefore proposing two alternative routes ((a) and (b), Figure 5) that from the intermediate bicyclic precursor generate the carbotricyclic carbon skeleton of FCs. Pathway (a) involves only one 1-2-hydride shift, resulting in the labeling of hydrogens bonded to C-3 and C-6, while pathway (b) involves two 1,2-hydride or 1,3-hydride shifts, resulting in the labeling of hydrogens bonded at C-6 and C-7. [35,54]. These two hypothesized cyclizations of the biosynthetic bicyclic intermediate are in agreement with the results reported by Barrow et al. [55], using NMR analysis after [1-^13^C], [2-^13^C]-acetate, and [3-^13^C]MVA incorporation into FC. ^13^C atoms were incorporated in positions 3, 7, 11, 15, and 24 of 19-deoxydideacetylFC (**9**) and FC-H (**13**), as ascertained by recording their ^13^C NMR spectra [56].

FC obtained by the incorporation of [(4*R*)-4-^3^H, 2-^14^C]-MVA had one tritium at C-6, found converting FC in the **29** derivative (Figure 3, Table 1), by catalytic hydrogenation of **1** [57], while any tritium atom was lost by C-3 when FC was converted in the 3-α-hydroxydideacetyl FC (**11**), by allylic hydroxylation and thus losing the proton at C-3 [25,35]. The same results were obtained by Banerji et al. [33] by the incorporation of [3-^13^C, 4-^2^H_2_]MVA in FC followed by ^13^C NMR, which allowed, using deuterium tracing, to localize ^13^C incorporation at C-5, C-7 and C-23, respectively [33]. Thus, the route (b) provides for final cyclization through two 1,2-or 1,3-hydride of the intermediate bicyclic precursor in the tricyclic carbon 5:8:5 skeleton of FCs.

In addition, the succession of functionalization events of the carbotricyclic skeleton of FC aglycone was studied. The synthesis of derivative **27** from labeled FC, obtained by the incorporation of [^14^C-methy]-L-methionine in **1,** and the methylation of the hydroxyl group at CH_2_-16 by *S*-adenosylmethionine, caused a total loss of radioactivity. Further studies were carried out incorporating in FC metabolites **3**, **9**, and **12** (Figure 1) having a tritium at C-8. The compounds were completely acetylated and oxidized with Jones reagent [58] in the corresponding polyacetyl-8-keto derivatives. The 8-keto derivatives were first reduced with tritium-labeled sodium borohydride (NaBH_4_)_,_ to obtain a tritium bonded at C-8, and then converted in the starting derivatives (**3**, **9** and **12**) by deacetylation. The radioactivity was completely lost when FC obtained from the incorporation of C-8 tritiated **3** and **9** was converted in **24** (Figure 1, Table 1) and its corresponding 19-deacetyl derivative, while it was retained when the same conversion was operated on FC obtained from the incorporation of C-8 tritiated **12** [54].

**Figure 6:**
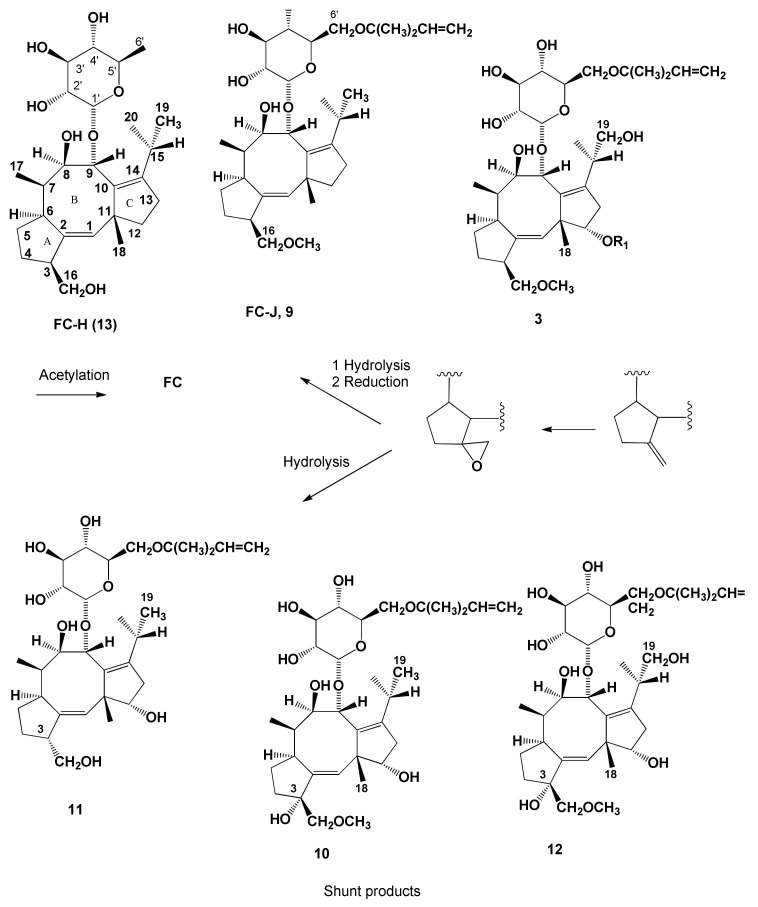
The successive steps of functionalization of the carbotricyclic 5:8:5 diterpenoid glucosylated intermediate in the FC biosynthesis, and related minor metabolites and shunt biosynthetic products.

These results demonstrated that **3** and **9** are precursors of FC while **12** is a shunt biosynthetic product (Figure 6). Moreover, it was independently demonstrated by Barrow et al. [23] that 19-deoxydideacetylFC (also named FC-J) (**9**) and FC-H (**13**) were precursors of **1**. These results allowed the abilty to hypothesize that, starting from FC-H (**13**), the *t*-pentenylation of the sugar moiety of FC or the methylation of the hydroxymethyl group at C-3 were followed by the hydroxylation of C-19 and finally by its acetylation and the acetylation of the hydroxy group at C-3′ (Figure 6). The minor metabolites **10**, **11** and **12** are shunt biosynthetic products and originated from conversion to the corresponding epoxide of the double bond between C-3 and C-16, followed by reduction, to give FC and minor metabolites belonging to the same subgroup. Metabolites belonging to the 3α-hydroxy subgroup originated from the hydrolysis of the same epoxide (Figure 6) [51,54].

Finally, the biosynthetic origin of the isopropyl group linked to C-14 was demonstrated by incorporating [2-^3^H_2_, 2-^14^C]-MVA into FC. The radioactive FC was converted into the corresponding acetonidedeacetylaglycone (**33** and this latter into the corresponding tetraene (**38**, Figure 2, Table 1). The tetraene was allylic hydroxylated at C-20 [52] affording derivative **41** (Figure 2, Table 1) and in turn oxidized to the corresponding α,β-unsaturated aldehyde. The loss of tritium radioactivity was 2/3 in the hydroxyl tetraene and 1/3 in the aldehyde. This finding demonstrated that the C-20 of FC originates from the C-2 of MVA [39].

More recently it was reported that, despite the classic known pathway for the biosynthesis of all diterpenes which originate from GGPP, the (+)-fusicocca-2,10(14)-diene, is a tricyclic hydrocarbon precursor for FCs. It was biosynthesized from the C-5 isoprene units by the unusual multifunctional enzyme *P. amygdali* fusicoccadiene synthase (PaFS), which shows both prenyltransferase and terpene cyclase activities [59].

## 2. FC: A Tool in Plant Physiology

### 2.1. Biological Activity

**Figure 7:**
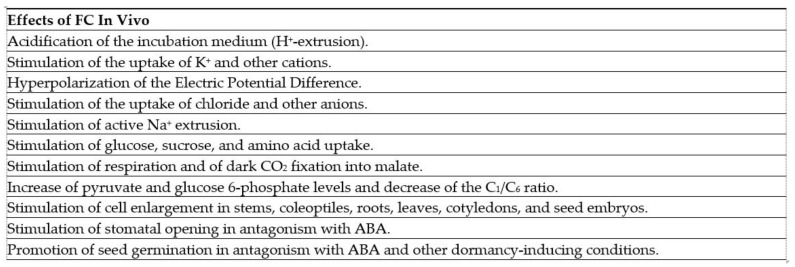
Reproduction of Table 1 of “Fusicoccin, a Tool in Plant Physiology” by Erasmo Marrè [61], reporting the main effects promoted by FC in vivo. ABA: absisic acid.

The remarkable phytotoxic activity of FC and the finding that it was even more effective than the growth-promoting hormone auxin in stimulating cell enlargement and tissue growth [60] prompted further investigation concerning its effects in plants. These studies followed the pilot-scale production of cultures of the fungus in 3000 L fermenters [50], that made large amounts of the toxin available for experimentation and led to the discovery of a number of physiological effects elicited by FC in higher plants. They included, at the tissue level, an increase of growth, the opening of stomata, nutrient uptake, and the breaking of seed dormancy [61]. At the cellular level, it was established that growth was accompanied by H^+^ extrusion, hyperpolarization of the plasma membrane potential, and K^+^ uptake; all effects very similar to those induced by auxin. Figure 7 reports Table 1 from the review “Fusicoccin: A Tool in Plant Physiology”, by E. Marrè, published in the Annual Review in Plant Physiology in 1979 [61], in which all the effects of FC in plants, known at that time, were summarized. This paper represents a milestone for FC research, since from those circumstantial pieces of evidence, Marrè proposed that the molecular basis underlying the FC action was the stimulation of the electrogenic proton pump of the plasma membrane of plant cells. This primary event produces acidification of the external medium and hyperpolarization of the plasma membrane potential, thereby providing the driving force for ion uptake, cellular transport, and cell enlargement. This model turned out to be substantially correct as it was confirmed by experimental work in the successive years. Following progress in the purification of plasma membranes, a detailed biochemical characterization of the FC-induced stimulation of the H^+^-ATPase was carried out in two-phase partitioned plasma membrane vesicles, where it was ascertained that FC brought about an increase of the Vmax of the enzyme, together with a shift in its pH optimum [15,16]. The conclusive demonstration that the H^+^-ATPase is the target of the toxin was obtained by the expression of the H^+^-ATPase C-terminus in yeast or by the complementation of the yeast H^+^-ATPase with the *Arabidopsis thaliana* AHA2 and *Nicotiana plumbaginifolia* PMA2 isoforms, which generated high affinity FC-binding sites [62,63,64].

### 2.2. Structure-Activity Relationships

The large scale production of FC [50] that was made available also to produce consistent amounts of several minor metabolites (see Section 1.2), as well as to prepare several semisynthetic derivatives, thus allowing the performance of structure/activity relationship studies. A list of metabolites and/or derivatives together with their corresponding biological activity is reported in Table 1.

**Figure 8:**
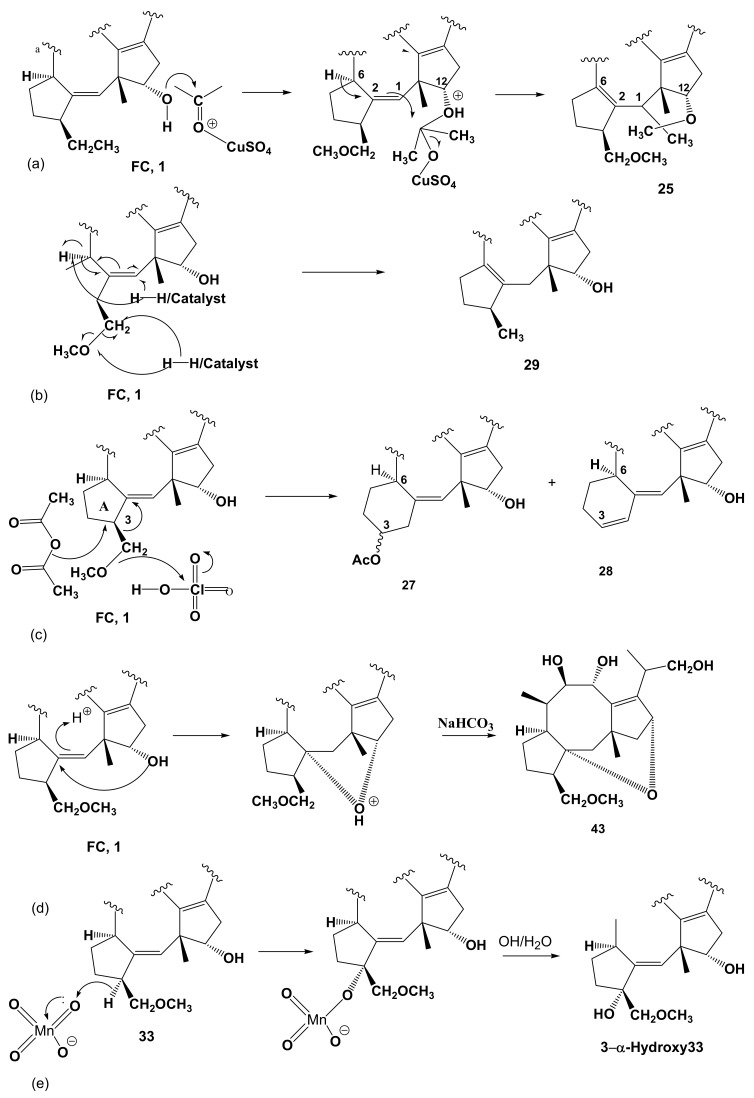
Unusual products obtained from FC derivatization. In (**a**–**e**) the detailed hypothesized mechanisms are shown.

The most unusual reaction products obtained from the derivatization of FC are reported below. In fact, by the reaction with anhydrous CusO_4_ in dry acetone that normally permits the conversion of a diol into the corresponding 2,2-dimethylketal, FC (**1**) gave the pseudoacetonide derivative **25** (Figure 3, Table 1) [30]. This derivative showed a pseudocetonide ring between C12 and C1 followed by the double bond shift from C1/C2 to C2/C6 according to the hypothesized reaction mechanism (a) as reported in Figure 8. By catalytic hydrogenation using 10% Pd/C, **1** was converted in derivative **29** [57] This compound not only showed the saturation of the *t*-pentenyl residue attached to C-6′ of glucose moiety into the corresponding *t*-pentyl, but also the hydrogenolysis of the methylenemethoxy group at C3, with a consequent shift of the double bond from C1/C2 to C2/C6, according to the hypothesized reaction mechanism (b) reported in Figure 8. The reaction of FC with the Fritz and Shenk reagent, normally used to acetylate tertiary or hindered hydroxyl groups [52], surprisingly produced, in addition to the expected acetylation of all the secondary hydroxy groups, but not the hindered HO-C(8), the enlargement of the cyclopentane ring A into the corresponding cyclohexane ring. This rearrangement occurred with the scission of the methylenemethoxy group and the acetoxylation of C-3, generating the derivative **27**. The successive β-elimination of acetic acid generated the diene **28** [32] according to the hypothesized mechanism (c) reported in Figure 8. The acid treatment of the deacetylaglycone of FC allowed it to obtain its unusual isomer ether **43** (Figure 3, Table 1), which showed the presence of an ethereal bridge between C-1/C12 generating a corresponding new tetrahydropyran ring [40]. The hypothesized mechanism (d) for the conversion of **1** into **43** is reported in Figure 8. Finally, the allylic hydroxylation operated by KMNO_4_ in alkaline conditions allowed it to convert the acetonide deacetylaglyconeFC **33** into the corresponding β-hydroxyderivative (**37**, Figure 3, Table 1) [26]. The same reaction carried out on the dihydroderivative of **3** allowed it to confirm the structure of **11** [26] according to the mechanism (e), reported on Figure 8.

Data listed in Table 1 allowed for the first time the ability to outline the structural requirements necessary for biological activities.

Considering the effect of FC on the physiological processes reviewed by Marrè [61] and listed in Figure 7, FC, its natural analogues **3**, **9**, **10**, **12**, **13** (Figure 2), its semisynthetic derivatives **17**, **26**, (Figure 2) **31** (Figure 3), **33** (Figure 3), 9-*epi*-dideacetylFC, aglycone of *9-epi*-FC, aglycone of 19-deoxydideacetylFC, cotylenol (**39**, Figure 3) and cotylenins A, C and E [37], were assayed on the growth by cell enlargement and on the proton extrusion in pea (*Pisum sativum* L.) internode segments, on growth by cell enlargement in isolated squash (*Cucurbita maxima* Duchesne) cotyledons, on germination of light-requiring lettuce (*Lactuca sativa* L.) and of abscisic acid-inhibited radish (*Raphanus sativus* L) seeds. Results demonstrated that the activity of FC is completely or largely retained in dihydroFC (**14**) and in the derivatives having a *t*-pentyl group instead of a *t*-pentenyl one attached to the sugar residue. Instead, other structural modifications, as well as the loss of one or both acetyl groups, always determine a strong decrease of activity. A similar behavior also was shown by minor metabolites **3**, **10**, **12**, **13**, by cotylenins A,B,C, and by the aglycones of 19-deoxydeacetyl-FC and of cotylenol. Overall, these results demonstrated that the main structural features necessary for the biological activity are the configuration at C-9, the derivatization of the OH groups at C-8 and C-9, and the unaltered stereochemistry of the FC aglycone. On the other hand, the hydroxy groups at C-12 and C-19, the hydrogen at C-3 (that can be substituted by OH), and the stereochemistry of this latter carbon, as well as the methylation at C-16, do not affect biological activity [17]. These results were in agreement with and further extended previous data obtained by testing a smaller number of FC derivatives on the growth of etiolated pea seedlings cut at the first node [10] and corroborated the hypothesis from Marrè [61] that the activity of FC on different physiological processes was dependent on its capability to activate a single system involved in proton extrusion. The relevance of the conformation of the carbotricyclic ring for biological activity was confirmed as well by high resolution ^1^H NMR spectroscopy studies carried out on FC and different active and inactive derivatives and analogues as compounds **12**, **29**, **31**, **39**, **43** and (7*S*,9*S*)-diastereomer of the FC aglycone [65].

Concerning phytotoxicity, modifications such as partial or full deacetylation or acetylation, removal of the *t*-pentenyl group, as well as removal of the glucosidic moiety, strongly or completely reduced the phytotoxicity on four-leafed cuttings from tomato plants, while derivatives obtained by the hydrogenation of the *t*-pentenyl to *t*-pentyl group partially retained the phytotoxic activity [10]. On the other hand, different results were observed when the same compounds were assayed on etiolated pea seedlings [11]. In this case, partially or totally deacetylated derivatives, such as dihydroFC (**14**, Figure 2, Table 1,) and de-*t*-pentenylFC (**17**, Figure 2, Table 1), were as active as FC, whereas the deacetylaglycone (**31**, Figure 3, Table 1,) resulted less toxic and the FC acetyl derivatives completely inactive. Semisynthetic derivatives, such as de-*t*-pentenylisoFC, de-*t*-pentenylalloFC, de-*t*-pentenyltriacetylFC, 3′-monodeacetylFC, 3′-monodeacetyldihydroFC, 19-monodeacetylFC, 19-monodeacetylisoFC, 19-monodeacetylalloFC, 19-monacetyldihydroFC, 19-monacetyldihydroisoFC, 19-monacetyldihydroalloFC, 12-acetyldideaceylFC, and 12-aceyldideactyldihydroFC assayed in comparison to **1**, **14**, **17** and **18** for toxicity on tomato cuttings, confirmed that the conversion of the *t*-pentenyl residue to *t*-pentyl increased phytotoxicity. Among the de-*t*-pentenyl derivatives, those having a different acyl group, or partially deacetylated derivatives, were all less toxic than the FC [12]. The diverse phytotoxicity of minor metabolites or derivatives on different tissues or plants may be partially explained in terms of the different polarity of the tested compounds, which can influence their bioavailability.

As far as the ability to induce abscission, FC and the semisynthetic derivative monodeacetyl-, dideacetyl-, iso-, de-*t*-pentenyl-FC (**2**, **3**, **4**, **17**, Figure 2, Table 1) monodeacetylde-*t*-pentenylFC and dihydro-, monodeacetyldihydro, dihydrodideacetyl-FC, (**13**, **19** and **20**, Figure 2, Table 1) were tested on the explants of mature leaves of *Citrus sinens* [13]. FC and derivatives **2** and **4** were the most active in accelerating abscission, a fact that is very likely due to the stimulation of ethylene synthesis observed in the same system [14]. From structure/activity relationships it was inferred that the effect on abscission was influenced mainly by the presence of the glucose moiety, whereas a minor contribution was also owed to the double bond of the *t*-pentenyl residue and the acetyl group at C-19.

Several natural analogues and derivatives of FC were tested as well as potential natural herbicides by exploiting the effect of FC on seed germination in order to induce on parasitic weeds, such as *Orobanche ramosa*, the so called “suicidal germination” [19]. They were compounds **3**, **8**-**11**, **13**, **17**, **20**, **24**, **25**, **27**, **28**, **31**, **33**, **34**, **43**, **46**, **47**, **49**, **50**, de-*t*-pentenyltriacetylFC [17], acetonide of 19-deoxydideactylFC [22] and cotylenol (**39**). Among them 16 were glucosides and 9 were aglycones. Results indicated that FC and cotylenol (**1** and **39**) were almost inactive and deacetylaglyconeFC poorly active, whereas 8,9-isopropylidenedeacetylaglyconeFC (**33**) and the dideacetylFC (**3**) were highly active. From these studies it was inferred that in both groups of glucosides and aglycones the most important structural feature for activity was the presence of the primary hydroxy group at C-19, while substituents and the overall conformation of the carbotricyclic ring also played a significant role [19].

### 2.3. Mode of Action in Plants

Taking advantage of the above reported structure/activity relationship studies, a tritiated dihydroFC ([^3^H]FC) (33,34-ditritiumFC), displaying the same activity of FC in different biological assays [66,67], was synthesized, thus allowing the ability to perform an investigation aimed to locate the target of FC in plant cells. FC-binding sites were detected for the first time by Dohrmann et al. [68] in the microsomal fraction of maize coleoptiles and subsequently in several plants [69,70,71] until, in a systematic study, it was ascertained that they are present in the plasma membrane of all plants, from liverworts to angiosperms [72]. Furthermore, [^3^H]FC binding studies allowed the characterization of different biochemical parameters of microsomal FC-binding sites from different sources, such as pH optimum [73], saturation range [69,70,72,74,75,76], specificity [17,67,77], and dissociation constants.

**Figure 9:**
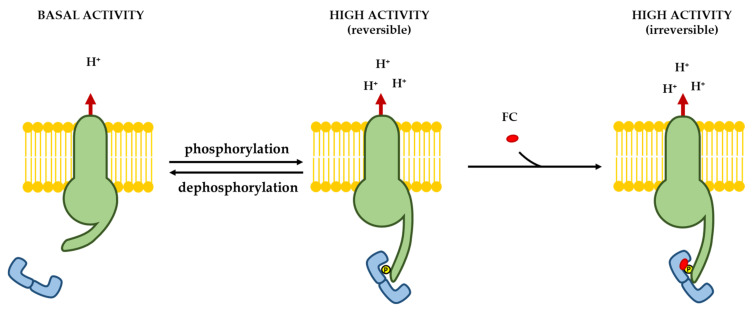
Activation of the plasma membrane H^+^-ATPase by 14-3-3 proteins and FC. Phosphorylation of the C-terminal penultimate Thr residue (Thr947 in the Arabidopsis H^+^-ATPase AHA2 isoform) generates a 14-3-3 binding site. Association of 14-3-3 proteins leads to the displacement of the C terminus of the proton pump and consequently to its activation. FC greatly stabilizes the interaction, causing irreversible activation of the H^+^-ATPase.

Attempts to purify the FC-binding sites remained unsuccessful for nearly twenty years, until the introduction of new, advanced chromatographic techniques allowed three groups to independently identify 14-3-3 proteins in highly enriched fractions of FC-binding sites from different sources [78,79,80]. 14-3-3s are a family of regulatory proteins widespread in eukaryotic organisms [81], whose structure and functions were at that time very poorly characterized in animals and practically unknown in plants. However, in successive years, an impressive body of evidence was accumulated demonstrating that 14-3-3 proteins participate in the regulation of a number of pivotal physiological processes, such as cell cycle progression, apoptosis, cellular trafficking, and gene transcription [82,83], and that they are also implicated in the etiology of different diseases, including cancer [84,85]. The discovery that they were involved in the mechanism of action of FC stimulated research in plants, where it was ascertained that they also regulate other peculiar processes, such as nitrogen and carbon metabolism [86], ion transport [87,88], as well as hormone and light signaling [89,90,91,92]. A further decisive breakthrough in the identification of FC receptors was obtained by purification experiments after in vivo administration of the toxin to plant tissues. In fact, in these studies a strict correlation between H^+^-ATPase stimulation and the association of 14-3-3 proteins to the plasma membrane [62,63,89,93,94,95,96,97] was observed, suggesting that the FC promoted the association of stimulatory 14-3-3 proteins to the plasma membrane H^+^-ATPase. The ultimate evidence that FC binds to the H^+^-ATPase/14-3-3 complex was obtained by interaction studies using a synthetic peptide reproducing the last part of the C-terminus of the H^+^-ATPase [98], or yeast cells expressing the plant H^+^-ATPase [63]. In accordance with the inferred model of action, the toxin irreversibly stabilizes the interaction of the 14-3-3 with the C-terminus of the H^+^-ATPase, an event that displaces the autoinhibitory C-terminal domain, thereby leading to the stimulation of the activity of the enzyme [63]. The model of the activation of the H^+^-ATPase by 14-3-3 and FC is shown in Figure 9.

Phosphorylation of the Thr residue within the C-terminal sequence -Tyr-Thr-Val-COOH (Thr947 in the AHA2 *Arabidopsis thaliana* H^+^-ATPase isoform) generates a 14-3-3 binding site. The binding of the 14-3-3 proteins leads to the displacement of the H^+^-ATPase C-terminus and consequently to enzyme activation. FC strongly stabilizes the interaction, bringing about the irreversible activation of the H^+^-ATPase.

Today it is well clarified that 14-3-3 proteins bind to phosphorylated client proteins, thereby modulating their subcellular localization, enzymatic activity, turn-over, or association with other proteins [82,84]. In fact, the structure of 14-3-3 proteins has been elucidated by X-ray crystallography [99,100,101], revealing that each monomer, composed by nine anti-parallel *α*-helices, associates through its *N*-terminus to a second monomer in the functional dimeric protein. The dimer has a characteristic W-like shape, with a conserved internal surface that contains a conserved amphipathic cavity, where the phosphorylated target sequence accommodates [90,102,103]. Originally, only two consensus binding motifs on 14-3-3 clients, containing a phosphorylated Ser or Thr and called mode I (RSX(pS/pT)XP and mode II (RXY/FX(pS/pT)XP, respectively [102], were known. Therefore, the finding that 14-3-3 proteins interacted with the H^+^-ATPase was initially rather surprising, since no canonical mode I or II binding motifs are present on the sequence of the enzyme. However, successive studies clarified that a novel 14-3-3 binding site, formed by the terminal sequence –YpTV-COOH, generated by phosphorylation of the Thr residue, occurred at the extreme end of the C-terminus of the enzyme [104,105]. FC strongly and irreversibly stabilizes the complex between the C-terminus of the H^+^-ATPase and 14-3-3 proteins. The molecular details concerning the interaction of FC with the H^+^-ATPase/14-3-3 complex were inferred from the resolution of the crystal structure of the ternary complex between FC, 14-3-3 proteins, and a phosphorylated C-terminal peptide of the H^+^-ATPase [106].

**Figure 10:**
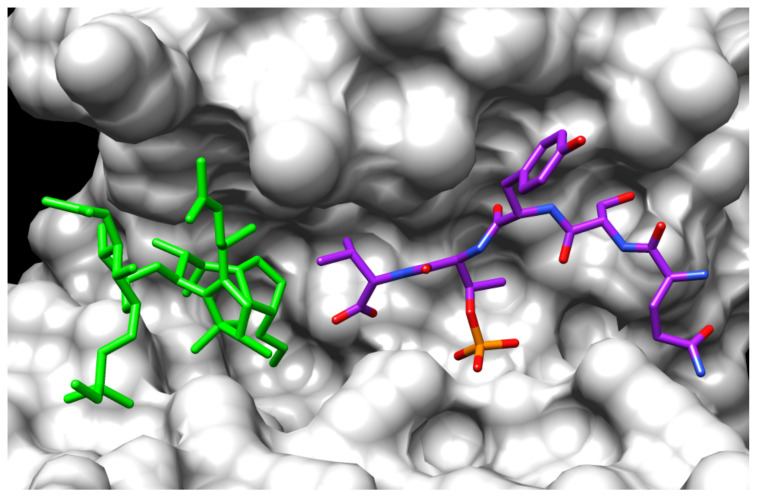
Crystal structure of the FC/phosphopeptide/14-3-3 ternary complex. Ternary complex between FC (green sticks), the H^+^-ATPase pentapeptide Gln-Ser-Tyr-phosphoThr-Val (purple stick) and 14-3-3 (grey surface). The interaction between the H^+^-ATPase peptide and FC involves the C-terminal valine of the peptide and the terpenoid moiety of FC. Molecular graphics were performed with UCSF Chimera, developed by the Resource for Biocomputing, Visualization, and Informatics at the University of California, San Francisco [106], using 1O9F pdb file.

It was ascertained that the phosphorylated peptide reproducing the conserved last five amino acids (QSYpTV-COOH) of the C-terminus is accommodated within the amphipathic binding groove of the 14-3-3 monomer, while the FC is arranged next to the C-terminus of the phosphopeptide, contacting both the valine residue of the peptide and the 14-3-3 groove, mainly by hydrophobic interactions and van der Waals contacts, through its diterpene moiety (Figure 10). By isothermal titration calorimetry (ITC) [107] and surface plasmon resonance [104], it was ascertained that FC binds 14-3-3 proteins with very low affinity (Kd FC/14-3-3, 50 µM), whereas the peptide and the toxin reciprocally enhance, by approximately two orders of magnitude, their respective binding affinities for 14-3-3, thus bringing about a strong stabilization of the ternary complex. This finding very likely underlies the previous characterization by Scatchard analysis of two classes of FC binding sites with very different affinities. Interestingly, crystal structure analysis corroborated previous results of structure/activity relationship studies obtained by Ballio et al. [67]. In fact, it was confirmed that even minor modifications of the carbocyclic ring impair biological activity as well as the ability to compete with tritiated FC in binding assays: epimerization in position C3 in ring A, or position C9 in ring B determines a complete loss of binding activity as does the introduction of a double bond between positions 2 and 6, altering the spatial orientation of ring A and B. On the other hand, more extensive changes onto the glycosidic part and the residual isoprene structure, such as the hydrogenation of the *t*-pentenyl moiety, or its complete removal or its derivatization, have no influence on binding [67]. The removal of the 3′ substituent has a minor effect, whereas the complete deletion of the glycosidic part results only in a reduced biological activity of FC, so that presumably, the glycosidic moiety influences predominantly the water solubility and/or bioavailability of the toxin, and therefore its phytotoxicity [17,65,66].

The identification of the 14-3-3 binding site of the proton pump was the first evidence of the occurrence of a novel, C-terminal 14-3-3 binding motif. Subsequent studies confirmed that, even though the majority of 14-3-3 clients bind 14-3-3s through mode I and II motifs, a reduced number binds through C-terminal sequences, called mode III motifs ((pS/pT)X1−2-COOH [108]. Crystallographic data allowed clarification that FC is ineffective on mode I and II interactions because it is unable to fit into the 14-3-3 binding groove that in these sequences is usually occupied by a Pro residue located at the +2 position with respect to the pSer residue [109,110,111].

## 3. FC: A 14-3-3-Targeted Drug

### 3.1. Stabilization of 14-3-3/Mode III-Client Interactions

Although it was initially believed that FC receptors were restricted to vascular plants [112], the finding that mode III 14-3-3 binding motifs are relatively widespread both in plant and animal proteomes raised the possibility that, in addition to the H^+^-ATPase/14-3-3 complex, other FC receptors could exist, thus prompting studies aimed to identify new FC receptors, as well as to unveil further effects of FC, especially in animals, where 14-3-3 proteins are known to regulate fundamental cellular processes involved in the etiology of various diseases, including cancer and neurodegenerative diseases [113,114,115]. The search in plants led to the identification of the K^+^ outward channel (KAT1) of *Arabidopsis thaliana* as a novel FC receptor. In fact, Saponaro et al. [116] ascertained that 14-3-3 proteins bind to KAT1 at its C-terminal site (YFSpSN-COOH), and that FC greatly stabilizes the interaction, thereby increasing the stimulatory effect of 14-3-3 proteins on K^+^ transport. Resolution of the crystal structure of the ternary complex showed that FC, owing to the steric hindrance of the C-terminal Asn, is forced in an unusual rotational conformation to fit the task within the binding site. In animals, different studies revealed that FC can influence diverse processes regulated by 14-3-3 proteins involving cell proliferation and differentiation and whose alteration underlies the development of various diseases. These emerging pieces of evidence are the premises to the exploitation of FC and/or of its chemical derivatives as 14-3-3-targeted drugs, able to stabilize the complexes between 14-3-3 and their clients, thereby inhibiting the signal transduction chain and resulting in a therapeutic effect. The first report regarding FC activity in animal cells was that by Bunney et al. [91], which showed that exposure to 50 µM FC of *Xenopus laevis* embryos altered their early phase of development, inducing randomization of the left-right axis of asymmetry (heterotaxia).The authors, by administration of a tritiated FC derivative to embryo tissue extracts and Scatchard analysis, demonstrated the occurrence of cytoplasmic binding sites for FC with affinity (Kd 27,8 nM) comparable to that of plant receptors. Further proof that the interaction of 14-3-3 with mode III targets can be stabilized by FC was obtained by Camoni et al. [109], who identified the platelet-adhesion receptor GPIb-IX-V as an FC receptor. The toxin stabilizes the association of 14-3-3s to Ibα, a glycoprotein part of the GPIb-IX-V complex, thereby stimulating the initial adhesion of circulating platelets to the sub-endothelial von Willebrand Factor (vWF). This finding opens the possibility of a therapeutic use of FC in genetic bleeding disorders, such as the Bernard-Soulier syndrome and the Glanzmann’s thrombastenia [117], as well as in anti-thrombotic drug development, since the interaction between vWF and GPIb-IX-V mediates the initial adhesion of platelets to the subendothelium in atherosclerotic vessels. 14-3-3 proteins are highly abundant in the central nervous system (CNS), where they function as pivotal regulators of axon development and guidance [118,119,120]. It has been demonstrated that FC stimulates in vitro damaged axon growth and in vivo regeneration of rat cortical embryonic neurons. Purification of cortical neuron lysates by affinity chromatography with immobilized FC allowed the identification of different proteins containing putative mode III binding motifs. Among them, the stress response regulator GCN1 was shown to form a complex with 14-3-3 proteins, which is stabilized by FC, thereby resulting in GCN1 turnover and neurite outgrowth and regeneration. It is known that the inability of the CNS to regenerate underlies persistent disability in spinal cord and nerve insult, therefore this finding suggests that FC may be a promising drug to target 14-3-3 protein-protein interactions to stimulate axon regrowth after injury, for CNS disease treatments [121]. In a successive study Kaplan et al. [122], using a new bioactive semisynthetic FC derivative (FC-NCPC; see Section 3.2.) with an improved affinity for 14-3-3 proteins, showed, in a mass-spectrometry/proteomics-based approach, that this compound can alter the 14-3-3 interactome of cortical neuron cell lysates, determining both the increase and decrease of association to the 14-3-3s of several clients. Bioinformatic analysis allowed the ability to ascertain that FC-NCPC treatment determined an enrichment in the interactome of components of the Rap1 signaling network [122]. These results suggest that the action of FC-NPC can be bifunctional, leading to the stabilization or disruption of its association with 14-3-3 proteins, depending on the client, and that FC-NCP stimulation of neurite outgrowth may depend on a polypharmacological mechanism, which involves the simultaneous modulation of the binding activity of 14-3-3 proteins participating in different signaling pathways. Well-documented evidence proves that 14-3-3 proteins are involved in the progression of various types of cancer [84,85], and that some FC-related terpenoids, such as cotylenin A [123] and ophiobolin A [124,125], possess anti-cancer activity, provided the rationale to test whether FC could affect cancer cells’ viability and proliferation. De Vries-van Leeuwen et al. [126] reported that FC induces apoptosis in various interferon-α-primed tumor cell lines. The same authors, in a successive study [127], showed that in tumor cells FC stabilizes the 14-3-3 complex with the estrogen receptor α (ERα). By co-crystallization of the ternary complex 14-3-3/ERα/FC, it was shown that phosphorylation of the penultimate C-terminal residue of the receptor generates a mode III 14-3-3 binding site sensitive to FC action. Complex stabilization hampers estradiol-induced receptor dimerization, which is a prerequisite to the signaling activity, leading to gene transcription and cell proliferation. Since deregulation of ERα activity is involved in breast cancer development and ERα is a target of breast cancer therapies, these results provide the rationale to a new approach based on the inhibition of receptor dimerization, mediated by protein/protein interaction stabilizers such as FC. Bury et al. [128] showed that FC reduces the proliferation of human glioblastoma multiforme (GBM) cells lines recalcitrant to pro-apoptotic stimuli, by increasing the duration of cell division. The migration ability was also reduced. At the structural level, changes in the actin cytoskeletal organization were observed, while at the molecular level it was demonstrated that FC decreased the activity of several protein kinases in vitro, including focal adhesion kinase (FAK), involved in cell proliferation and migration. These results suggest that FC may be used as a chemical template to develop toxin derivatives for GMB therapy.

Paiardini et al. [111] carried out a systematic in vitro analysis of the effect of FC on the binding between 14-3-3 proteins and phosphopeptides containing known mode III binding sequences of human proteins. By ITC and bioinformatic analysis the phosphopeptide structural requisites necessary for FC binding to the binary complexes were assessed. It was determined that the occurrence of a hydrophobic side chain residue in +1 position in respect to pSer/pThr strongly favors the interaction, whereas cyclic rings-containing residues hamper the assembly of the ternary complex, due to steric hindrance preventing the accommodation of FC in the 14-3-3-binding cavity. A new insight into the FC stabilizing effect on 14-3-3/client interactions was provided by the study of Stevers et al. [129], concerning the cystic fibrosis transmembrane conductance regulator (CFTR), which led to the identification of a novel non-canonical, FC sensitive, 14-3-3 binding sequence. The interaction of CFTR with 14-3-3 proteins is regulated by two diverse phosphorylated motifs which are accommodated simultaneously into the two binding grooves of a 14-3-3 dimer, with different affinities. The weaker affinity motif, (RIpSer^753^VIS), although it does not resemble either a mode I/mode II or a C-terminal mode III binding sequence, can be stabilized by FC. Crystallographic data allowed the determination that FC interacts with Val and Ile in position +1 and +2 to the pSer, respectively. It was also shown that FC binding stimulates the trafficking to the plasma membrane of a deleted form of CFTR (F508del-CFTR). In cystic fibrosis two classes of mutations affect the expression of CFTR in epithelial cells, resulting in reduced chloride and bicarbonate fluxes across the plasma membrane. Most diffused are class II mutations which affect the delivery to the plasma membrane of deleted but still functional forms of CFTRs. Hence, these results lead to envisage the druggability by FC or its derivatives of the 14-3-3/CFTR complex as a new approach for cystic fibrosis therapeutics development.

### 3.2. FC Derivatives for Pharmacological Applications

Several semisynthetic derivatives were prepared in the early years of FC research during systematic studies aimed to clarify structure, biosynthesis, and mode of action in plants of the phytotoxin. The chemical structures of the main derivatives obtained are shown in Figure 2 and Figure 3. These semisynthetic analogues are still an outstanding example of fine chemical modifications carried out on a complex diterpenoid. Their production, together with the crystallographic elucidation of the molecular basis of the interaction between FC and 14-3-3/clients complexes, provided the background necessary to prepare further derivatives, in more recent studies aimed to develop 14-3-3-targeted drugs.

**Figure 11:**
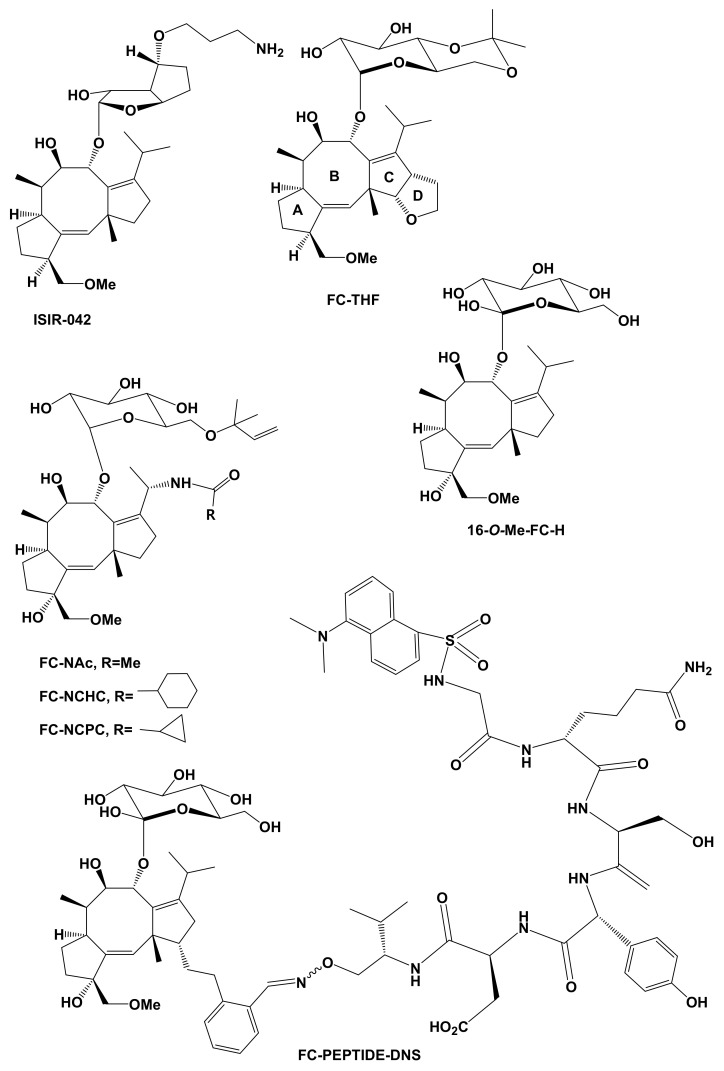
Semisynthetic FC derivatives for pharmacological applications.

To this purpose, different groups investigated the possibility to introduce chemical modifications in the fusicoccane carbon skeleton, to produce semisynthetic derivatives with improved affinity for 14-3-3 proteins. Indirect evidence of the druggability of FC came from studies concerning cotylenin A (Figure 2). Cotylenin A was originally shown to induce monocyte differentiation of human myeloma cell lines [130] and successively to display various other activities towards different types of cancer cells [130,131,132,133,134]. Crystallographic analysis allowed the ability to ascertain that cotylenin A also stabilizes mode I and II client/14-3-3 interactions. This capability was related to the lack in cotylenin A of the hydroxyl group at C-12 as compared to FC [135,136,137] and provided a rationale for its apparent higher cytotoxicity towards cancer cells. This hypothesis was later confirmed by Ohkanda et al. [138] in a structure/activity relationship study based on an in vitro assay of the fluorescent labeling of 14-3-3 proteins. Two fluorescent semisynthetic FC derivatives possessing or not the C-12 hydroxyl group, respectively, were tested for their capability to label 14-3-3ζ, forming ternary complexes in presence of phosphorylated hexapeptides reproducing mode I (RSHpSXP) or pentapeptides reproducing mode III (RSHpSX) motifs. Results demonstrated that the C-12 hydroxyl group hampers binding of FC to phospholigands having any amino acid at the +2 position, probably due to steric hindrance or unfavorable hydration. It was also demonstrated that only FC derivatives lacking the C-12 hydroxyl group showed significant cytotoxicity towards HEK293 and HL-60 cell lines. The first semisynthetic FC derivative produced with the aim to be used as an alternative or coadjuvant anti-cancer drug was ISIR-042, (Figure 11) whose structure is strictly related to that of cotylenin A, the main differences being the absence of the 3α-hydroxy group and the structure of the sugar moiety, where a -NH_2_ group was introduced to increase solubility. ISIR-042 inhibited pancreatic tumor cell growth under hypoxic conditions, an effect correlated to a reduction of HIF-1α accumulation and Akt activation [139]. It was also demonstrated that ISIR-042, in combination with tamoxifen and 5-fluorouracil or gemcitabine, was effective in overcoming the resistance to therapy of pancreatic cancer cells [140].

A semisynthetic FC derivative, namely FC-THF (Figure 11), with improved selectivity towards mode III motifs was designed by Anders et al. [141], by introducing a tetrahydrofuran ring at the C-12 of FC-J, the above cited natural analogue of FC **9**. In fact, although the inability of FC to stabilize canonical mode I and II interactions is due to the steric repulsion between the hydroxyl group at C-12 of the C ring and the conserved proline at the +2 position of binding sequences [129,135], many known 14-3-3 clients display non-canonical motifs, lacking the proline at the +2 position. Introduction of the tetrahydrofuran ring extended steric hindrance beyond the +2 position, thereby improving selectivity toward mode III motifs. FC-THF was shown to enhance the affinity of 14-3-3σ for a synthetic hexapeptide containing the 14-3-3 interaction site (369KRRK-pS-V374-COOH) of TASK-3, a human K^+^ channel expressed in neurons and other cell types. Andrei et al. [142], relying on the crystallographic information concerning the 14-3-3s/TASK3 hexapeptide interaction and using a molecular dynamics approach, rationally designed a semisynthetic FC derivatives with improved potency as a stabilizer of 14-3-3/client interactions. In this derivative named FC-NAc (Figure 11), besides the removal of the 3-acetyl-group, the 19-acetoxy group was substituted by an acetoamide group, which provided a hydrogen forming an extra hydrogen bond with the D215 residue within the binding groove of the 14-3-3s. Kaplan et al. [122] demonstrated that FC-NAc, as well as other 19 acetamide FC derivatives (FC-NCHC: N-cyclohexylcarbonyl derivative; FC-NCPC, N-cyclopropylcarbonyl, (Figure 11) are potent stimulators of neuron outgrowth, while proteomic data suggested that these compounds exert their effects by a polypharmacological mechanism, i.e., by at the same time reinforcing or disrupting the interactions of 14-3-3 proteins with different targets (see Section 3.1). The inhibition of 14-3-3/target interactions can be a promising pharmaceutical approach in some cancer types associated with an increased expression of 14-3-3 proteins [85]. Maki et al. [143] used a 14-3-3 guided ligation to link a C-19 epoxide-derivatized FC to a synthetic peptide containing a classical mode III motif (QSYpTV(FC-PEPTIDE-DNS, Figure 11). By ITC, it was shown that the introduction of the FC molecule greatly stabilized the interaction of the mode III peptide with the 14-3-3 binding groove, thereby suggesting that FC-linked peptides could be used as inhibitors of the binding of 14-3-3s to client proteins. This possibility has been investigated by Parvaktar et al. [144], who were able to generate FC-linked peptides intracellularly by oxime ligation between an FC derivative containing an *O*-formyl benzyl group at C-12 and a QSYpTV peptide containing an oxyamino group at the C-terminus. The intracellularly generated FC-linked peptide disrupted endogenous 14-3-3/cRaf interactions and provoked cell death, thus suggesting that the cytotoxicity was due to the inhibition of the association 14-3-3 proteins to clients. More recently Masuda et al. [145] synthesized an FC-conjugated peptide by a 14-3-3 guided, copper-free Huisgen cycloaddition, between a peptide fragment containing a 4,8- diazacyclononyne (DACN) moiety and an azide-containing FC derivative. The obtained derivative exhibited a moderate cytotoxicity towards colon cancer cells. Ca^2+^/calmodulin-dependent protein kinase kinase 2 (CaMKK2) is a 14-3-3-regulated, promising target for therapy of different diseases like obesity, diabetes and cancer [146,147]. Besides a canonical mode III motif located in the C terminal region, the protein contains an additional non-canonical 14-3-3 motif, which presents a Gln residue at the +2 position (RKLpS^100^LQE). Lentini Santo et al. [148] reported that the FC derivatives called FC-H and 16-O-Me FC-H (Figure 11), lacking substitutions in the C ring, stabilized the interaction between the *N*-terminal non canonical mode III motif, whereas FC, FC-J, and FC-THF which carry substituents at the C ring were ineffective, due to steric hindrance with the Gln residue of the peptide within the binding groove. Finally, in a study aimed to assess 14-3-3 isoform specificity of FC association to mode III targets, Sengupta et al. [149] tested the binding of different recombinant human 14-3-3 isoforms to phosphorylated hexapeptides containing known mode III motifs. Despite that isoform-dependent interactions were displayed better in the absence of FC, the stabilization by FC of the association of the 14-3-3σ isoform was apparently sensitive to the type of client peptide. Since 14-3-3σ is involved in cell death signaling and its expression is suppressed in various type of cancers, this finding is intriguing, leaving to envisage the possibility to rationally design isoform-specific synthetic FC derivatives for therapeutic purposes.

## 4. Conclusions

The history of FC began with studies regarding the canker disease induced by the fungus *P. amygdali* on almond and peach trees, which caused severe economic losses in the south of Italy in the 1960s. It is a story worth reviewing as it represents an outstanding example of technologically innovative, interdisciplinary, as well as integrated basic and applicative research that has lasted for sixty years and still is in progress, producing new and fruitful developments. The elucidation of the structure of the toxin involved a strict collaboration between plant pathologists and physiologists, chemists of organic natural substances, biochemists and structural chemists, as well as the extensive use of at that time very new techniques, such as mass spectrometry and NMR. The discovery that phytotoxicity was due to the ability of FC to irreversibly open stomata in all higher plants, despite the fungus is a pathogen of a restricted number of plant species, fueled studies aimed to discover the general process underlying FC effects in plant cells, which ultimately resulted in the ability of the toxin to stimulate the H^+^-ATPase of the plasma membrane, the master enzyme for the regulation of ion and nutrient transport in plants. This finding actually turned FC into a general tool in plant physiology for the investigation of transport and related processes and prompted at the same time early structure/activity relationship studies relying on the fine chemical synthesis of a number of semisynthetic FC derivatives, which allowed the ability to define basic structural requirements necessary to FC action. The identification of FC receptors in plants represented a further pivotal point of FC research. The discovery that 14-3-3 proteins are involved in the mechanism of stimulation of the H^+^-ATPase, being their association to the proton pump strongly stabilized by the toxin, opened new perspectives since 14-3-3 are a class of regulatory proteins ubiquitary in eukaryotes where they regulate fundamental cellular processes whose degeneration often underlies cancer or neurodegenerative disorders. Successive crystallographic analysis-based investigations, revealing that FC stabilizes only the interaction of 14-3-3s with clients possessing C-terminal mode III binding motifs, definitely focused applicative research to the identification of mode III targets involved in the etiology of different diseases and to the synthesis of new FC semisynthetic derivatives with improved affinity and/or selectivity, to be used as potential drugs in an innovative pharmacological approach based on small-molecule stabilization of protein-protein interactions, an emerging field of research in which the surprising story of FC continues to be written.

## Figures and Tables

**Table 1:** List of biological activities of FC and of minor diterpenoid metabolites from *P. amygdali* and FC semisynthetic derivatives used to carry out structure-activity relationships and mode of action studies. MFRM: minor FC-related metabolite; SFD: semisynthetic FC derivative; CM: *Cladosporium* sp. 501 7W metabolite; NA = Any activity; NT = Not tested.

Fusicoccanes	Relation to FC	Biological Activity	References
FC (**1**, Figure 1)		Phytotoxicity	[7,8,9,10,11,12]
		Induction of abscission	[13,14]
		Proton extrusion, potassium uptake and stomatal opening	[15,16]
		Cell enlargement, proton extrusion, cotyledon growth and seed germination	[17]
		Stimulation of seed germination	[18]
MonodeacetylFC (**2**, Figure 2)	MFRM	Reduced phytotoxicity	[2,9,10]
		Induction of abscission	[13,14]
DideacetylFC (**3**, Figure 2)	MFRM	Reduced Phytotoxicity	[2,9,10]
		Cell enlargement, proton extrusion, cotyledon growth and seed germination	[17]
		Induction of abscission	[13,14]
		Stimulation of *O. ramosa* seed germination	[19]
IsoFC (**4**, Figure 2)	MFRM	NT	[2,9,20]
		Induction of abscission	[13,14]
AlloFC (**5**, Figure 2)	MFRM	NT	[2,9,20]
MonodeacetylalloFC (**6**, Figure 2)	MFRM	NT	[21]
MonodeacetylisoFC (**7**, Figure 2)	MFRM	NT	[21]
12-AcetyldideacetylFC (**8**, Figure 2)	MFRM	NT	[21]
		Stimulation of *O. ramosa* seed germination	[19]
19-DeoxydideacetylFC (**9**, Figure 2)	MFRM	Cell enlargement, proton extrusion, cotyledon growth and seed germination	[17,22,23]
		Stimulation of *O. ramosa* seed germination	[19]
3-α-Hydroxy-19-deoxydideacetylFC (**10**, Figure 2)	MFRM	Cell enlargement, proton extrusion, cotyledon growth and seed germination	[17,24]
		Stimulation of *O. ramosa* seed germination	[19]
3-α−HydroxydideacetylFC (**11**, Figure 2)	MFRM	Cell enlargement, proton extrusion, cotyledon growth and seed germination	[17,25,26]
		Stimulation of *O. ramosa* seed germination	[19]
16-O-demethyl-19-deoxydideacetyl-3-*epi*-FC (**12**, Figure 2)	MFRM	Reduced phytotoxicity	[10,27,28]
		Cell enlargement, proton extrusion, cotyledon growth and seed germination	[17]
FC-H (**13**, Figure 2)	MFRM	Reduced phytotoxicity	[23]
		Cell enlargement, proton extrusion, cotyledon growth and seed germination	[10]
		Stimulation *of O. ramosa* seed germination	[19]
DihydroFC (**14**, Figure 2)	SFD	Phytotoxicity	[10,12,29]
		Induction of abscission	[12,13]
TriacetylFC (**15**, Figure 2)	SFD	NA	[10,29]
De-*t*-pentenyltetracetylFC (**16**, Figure 2)	SFD	Reduced phytotoxicity	[10,12,29]
		Stimulation of *O. ramosa* seed germination	[19]
De-*t*-pentenylFC, (**17**, Figure 2)	SFD	Reduced phytotoxicity	[10,12,29]
		Induction of abscission	[13,14]
		Stimulation of *O. ramosa* seed germination	[19]
IsodihydroFC (**18**, Figure 2)	SFD	NA	[10,12,29]
MonodeacetyldihydroFC (**19**, Figure 2)	SFD	Reduced phytoxicity	[10,29]
		Induction of abscission	[11,12]
DihydrodidacetylFC (**20**, Figure 2)	SFD	Phytotoxicity	[10,29]
		Induction of abscission	[13,14]
		Stimulation of *O. ramosa* seed germination	[19]
TriacetyldihydroFC (**21**, Figure 2)	SFD	No phytotoxicity	[10,29]
Dideacetyl-de-*t*-pentenylFC (**22**, Figure 2)	SFD	Reduced phytotoxicity	[10,29]
Triacetyl-de-*t*-pentenylFC (**23**, Figure 2)	SFD	NA	[10,29]
8-Oxo-triacetylFC (**24**, Figure 2)	SFD	NT	[13,29]
PseudoacetonideFC (**25** Figure 2)	SFD	NT	[29,30]
		Stimulation of *O. ramosa* seed germination	[19]
8-oxo-9-*epi*-dideacetylFC (**26**, Figure 2)	SFD	NT	[17]
Derivative **27** (Figure 2)	SFD	NT	[10,31]
		Stimulation of *O. ramosa* seed germination	[19]
Derivative **28** (Figure 2)	SFD	NT	[10,32]
		Stimulation of *O. ramosa* seed germination	[19]
PerhydroFC (**29**, Figure 3)	SFD	NT	[33]
		Stimulation of *O. ramosa* seed germination	[19]
De-t-pentenylperhydroFC (**30**, Figure 3)	SFD	NT	[33]
DeacetylaglyconeFC (**32**, Figure 3)	SFD	Reduced phytotoxicity	[7,10]
		Stimulation of seed germination	[18]
		Stimulation of *O. ramosa* seed germination	[19]
TetracetyldeacetylaglyconeFC (**32**, Figure 3)	SFD	No phytotoxicity	[7,10,34]
		Cell enlargement, proton extrusion, cotyledon growth and seed germination	[13]
8,9-IsopropylidenedeacetylaglyconeFC= 8,9-acetonideacetylglyconeFC (**33**, Figure 3)	SFD	NT	[7,34]
		No phytotoxicity	[10]
		Stimulation of *O. ramosa* seed germination	[19]
19-Trytil-8,9-cetonidedeacetylaglyconeFC (**34**, Figure 3)	SFD	No phytotoxicity	[10]
		Stimulation of *O. ramosa* seed germination	[19]
12-Oxo-19-trytil-8,9-actonidedeacetylaglyconeFC (**35**, Figure 3)	SFD	NT	[7,34]
12,19-Dimesyla-8,9-cetonidedeacetylagllyconeFC (**36**, Figure 3)	SFD	NT	[7,34,35]
3-α-Hydroxy-8,9-acetonidedacetylaglycone FC (**37**, Figure 3)	SFD	NT	[26]
Tetraene of 8,9-acetonidedeacetylaglyconeFC (**38**, Figure 3)	SFD	NT	[35]
Cotylenol (**39**, Figure 3)	CM	Cell enlargement, proton extrusion, cotyledon growth and seed germination	[17,36,37,38]
		Stimulation of seed germination	[18]
3α-Hydroxy-12,19-dimesyl-8,9-acetonidedeacetylaglyconeFC (**40**, Figure 3)	SFD	NT	[39]
20-Hydroxytetrahene (**41**, Figure 3)	SFD	NT	[39]
8,9-Acetonide cotylenol (**42**, Figure 3)	SFD	NT	[39]
Isomer of deacetylaglycone of FC (**43**, Figure 3)	SFD	NT	[40]
		Stimulation of *O. ramosa* seed germination	[19]
MonoaldehydedihydropyrandeacetylaglyconeFC (**44**, Figure 3)	SFD	NT	[34]
19-TosyldideactylFC (**45**, Figure 3)	SFD	NT	[19]
19-Fluoro-19-dehydroxydideacetylFC (**46**, Figure 3)	SFD	Stimulation of *O. ramosa* seed germination	[19]
AglyconeFC (**47**, Figure 3)	SFD	Stimulation of *O. ramosa* seed germination	[19]
19-Deoxy-12-*epi*-8,9-acetonide of deacetylaglycone FC (**48**, Figure 3)	SFD	Stimulation of *O. ramosa* seed germination	[19]
19-Deoxy-12-oxo-8,9-acetonide of deacetylaglycone FC (**49**, Figure 3)	SFD	Stimulation of *O. ramosa* seed germination	[19]
De-*t*-pentenyl-9-epi-tetracetylFC (**50**, Figure 3)	SFD	No phytotoxicity	[10]

## Data Availability

There are no supporting data.
